# Design of an RCT on cost-effectiveness of group schema therapy versus individual schema therapy for patients with Cluster-C personality disorder: the QUEST-CLC study protocol

**DOI:** 10.1186/s12888-022-04248-9

**Published:** 2022-10-08

**Authors:** Iuno Z. Groot, Anne-Sophie S. M. Venhuizen, Nathan Bachrach, Simone Walhout, Bregje de Moor, Kasper Nikkels, Susanne Dalmeijer, Myrte Maarschalkerweerd, Joël R. van Aalderen, Hinde de Lange, Renske Wichers, Agatha Ph. Hollander, Silvia M. A. A. Evers, Raoul P. P. P. Grasman, Arnoud Arntz

**Affiliations:** 1 Department of Clinical Psychology, University of Amsterdam, PO Box 15933, Amsterdam, 1001 NK the Netherlands; 2 Department of medical and clinical psychology, Tilburg University, Tilburg, the Netherlands; 3 GGZ-Oost Brabant, Department of Personality Disorders, Helmond, Boxmeer, Oss, the Netherlands; 4 PsyQ Amsterdam, Amsterdam, the Netherlands; 5 PsyQ Zaandam, Zaandam, the Netherlands; 6 PsyQ Rotterdam, Rotterdam, the Netherlands; 7 PsyQ Utrecht, Utrecht, the Netherlands; 8 Emergis, Goes, Netherlands; 9 IPGGZ Veendam, Veendam, the Netherlands; 10 IPGGZ Groningen, Groningen, the Netherlands; 11 Department of Health Services Research, Care and Public Health Research Institute (CAPHRI) Maastricht University, Maastricht, the Netherlands; 12 Centre for Economic Evaluation, Trimbos Institute, Utrecht, the Netherlands; 13 Department of Psychological Methods, University of Amsterdam, Amsterdam, the Netherlands

**Keywords:** Cluster-C personality disorder, Randomized controlled trial, Group schema therapy, Individual schema therapy, Economic evaluation, Personalized care

## Abstract

**Background:**

Given the high prevalence of Cluster-C Personality Disorders (PDs) in clinical populations, disease burden, high societal costs and poor prognosis of comorbid disorders, a major gain in health care can be achieved if Cluster-C PDs are adequately treated. The only controlled cost-effectiveness study published so far found Individual Schema Therapy (IST) to be superior to Treatment as Usual (TAU). Group ST (GST) might improve cost-effectiveness as larger numbers can be treated in (>50%) less time compared to IST. However, to date there is no RCT supporting its (cost-) effectiveness. The overall aim of this study is to assess the evidence for GST for Cluster-C PDs and to improve treatment allocation for individual patients. Three main questions are addressed: 1) Is GST for Cluster-C PDs (cost-)effective compared to TAU? 2) Is GST for Cluster-C PDs (cost-) effective compared to IST? 3) Which patient-characteristics predict better response to GST, IST, or TAU?

**Methods:**

In a multicenter RCT, the treatment conditions GST, IST, and TAU are compared in 378 Cluster-C PD patients within 10 sites. GST and IST follow treatment protocols and are completed within 1 year. TAU is the optimal alternative treatment available at the site according to regular procedures. Severity of the Cluster-C PD is the primary outcome, assessed with clinical interviews by independent raters blind for treatment. Functioning and wellbeing are important secondary outcomes. Assessments take place at week 0 (baseline), 17 (mid-GST), 34 (post-GST), 51 (post-booster sessions of GST), and 2 years (FU). Patient characteristics predicting better response to a specific treatment are studied, e.g., childhood trauma, autistic features, and introversion. A tool supporting patients and clinicians in matching treatment to patient will be developed. An economic evaluation investigates the cost-effectiveness and cost-utility from a societal perspective. A process evaluation by qualitative methods explores experiences of participants, loved ones and therapists regarding recovery, quality of life, and improving treatment.

**Discussion:**

This study will determine the (cost-)effectiveness of treatments for Cluster-C PDs regarding treatment type as well as optimal matching of patient to treatment and deliver insight into which aspects help Cluster-C-PD patients recover and create a fulfilling life.

**Trial registration:**

Dutch Trial Register: NL9209. Registered on 28-01-2021,

**Supplementary Information:**

The online version contains supplementary material available at 10.1186/s12888-022-04248-9.

## Administrative information

**Table:** 

Title	Design of an RCT on cost-effectiveness of group schema therapy versus individual schema therapy for patients with Cluster-C personality disorder: The QUEST-CLC Study Protocol
Trial register	Dutch Trial Register: NL9209. Registered on 28-01-2021
Issue date	29-06-2022
Protocol version	1
Funding	This trial has received funding from ZonMW (Dutch Organisation for Scientific Research) and participating mental health care institutes.
Role of sponsor	The funding source ZonMW has no role in the study design, the collection analysis or interpretation of the results.
Name and contact information for the trial sponsor	The principal investigators of this trial are prof. dr. Arnoud Arntz; (A.R.Arntz@uva.nl; Department of Clinical Psychology, University of Amsterdam; P.O. Box 15933, 1001 NK Amsterdam, the Netherlands) and dr. Nathan Bachrach.
Role of sponsor	The principal investigators have initiated this trial and have developed the study protocol in collaboration with the study board.

## Background

Cluster-C personality disorders (PDs; avoidant, dependent, and obsessive-compulsive) are characterized by severe anxiety, (interpersonal) dysfunction and maladaptive coping in dealing with PD-specific fears, such as avoidance and excessive control. The prevalence of Cluster-C PDs is 6.7% in the Western adult population [1] and 5% in the global population [2]. In clinical samples, Cluster-C PDs are estimated at a prevalence between 10.5% (for obsessive compulsive) and 24.6% (for avoidant) [3], and are associated with high comorbidity, notably social anxiety, eating disorders and depression [4].

Unsurprisingly, Cluster-C PDs impair quality of life, visible in decreased psychological well-being and impaired social relationships [5]. It has been found that individuals with Cluster-C PDs are impeded in marital status, educational achievements, and are more often economically inactive than individuals without PD [6, 7]. The latter is reflected in the fact that PDs are related to high economic costs in comparison to for instance depression or generalized anxiety disorder [8]. The annual cost of PDs in Europe is estimated at 27.3 billion euros [9]. For Cluster-C PD costs in the Netherlands are estimated between 12,000 and 13,500 euros per person per year (in 2007 prices) [10] and consist of elevated health costs, patient and family costs, productivity losses, and costs in other sectors. The total economic losses for this population may be even higher considering the difficulty to assess certain costs, such as loss of production due to excessive perfectionism, problems in cooperating with colleagues, and difficulties with fulfilling the role of parent [4].

Despite the high prevalence and the severe impact of Cluster-C PDs on individuals and society, there is a relative lack of attention in the scientific field to Cluster-C PDs in general and their treatment in particular. This scarcity of research on Cluster-C PDs is in part responsible for a lack of international guidelines concerning its treatment [11]. The absence of clear guidelines for adequate treatment might explain why the average number of previous treatments for Cluster-C PD patients was 2.33, with an average total duration of 2.67 years in one previous study on individual Schema Therapy (IST [12];). In addition, having a comorbid Cluster-C PD negatively affects the prognosis for other disorders and strongly predicts future functioning [4]. Considering the serious mental distress, dysfunction, and societal costs associated with Cluster-C PDs, more research into effective treatment is vital.

So far, the only treatment for Cluster-C PDs of which the (cost-)effectiveness was appropriately investigated (i.e., with a randomized controlled trial; RCT) is IST. A large Dutch RCT demonstrated superiority of IST to Treatment as Usual (TAU) for 6 PDs, of which >90% were Cluster-C [10, 12]. One year after completing IST, approximately 80% of patients recovered from their PD. ST assumes that maladaptive schemas can arise when a child's basic needs are not met and that these schemas underlie personality pathology [13, 14]. Central to contemporary ST are schema modes, which are defined as momentary states of mind which control the individual’s thinking, feeling, and acting at that moment. Dysfunctional schema modes can arise through the combination of maladaptive schemas and coping style and often had a functional role in childhood but became maladaptive in adulthood. Through the use of the therapeutic relationship (limited reparenting) and different techniques (including experiential ones), ST aims to reduce maladaptive and strengthen functional schemas and schema modes. Frameworks and specific schema mode models are available for Cluster-C PDs, showing the most common schema modes for avoidant, dependent and obsessive-compulsive PD, and the associated recommended treatment strategy [15].

A popular and economically attractive form of ST is group treatment, as larger numbers can be treated in less therapist time than with individual therapy (>50%), making it potentially more cost-effective than IST. In addition, the group format might offer extra therapeutic advantages such as support and understanding from group members which can help patients gain a sense of belonging and can facilitate secure attachment. Possibly group therapy would be exposure in itself for patients with cluster-c PD, where social avoidance and dependence play a major role in their pattern of symptoms. Furthermore, group therapy can induce vicarious learning and offer in vivo opportunities to practice with setting boundaries and expressing one's needs in a healthy way [16]. Group schema therapy (GST) has proven to be an effective treatment for borderline PD [17, 18] and was found superior to TAU [19, 20]. Only two studies have investigated GST for Cluster-C PD. First, a modest pilot study of a GST-protocol including 8 patients with mixed personality disorders (6 patients with avoidant PD and 2 with borderline PD) showed significant decline in avoidant PD symptom severity at follow-up [21]. The two patients with borderline PD were both reported as dropout, suggesting that mixing different PDs within this GST-protocol may be contra indicative. Second, a larger pilot study investigated our current GST-protocol in a multicenter open trial (9 sites, 137 patients). The treatment was highly acceptable: only 16 dropouts (11.6%). The decrease in symptoms from baseline to follow-up of the primary PD was large (effect sizes ranged from cohen’s d = .59 for obsessive compulsive PD, d = 1.33 for avoidant PD and, d = 1.52 for dependent PD).

It is likely that some patients are better off with individual (schema) therapy compared to group ST or TAU. Matching treatment to patient (personalized care) also reduces societal costs and demoralization in patients who are not helped by their treatment. Consequently, it is important to disentangle which variables do, and which do not, indicate which treatment is better for whom. Apart from demographic variables (e.g., age, gender), 4 potential predictors of treatment effect are identified: introversion, autism, trauma, and sleep. Introversion has been mentioned by client platform MIND [22] as a factor that might interfere with group therapy and should therefore be taken into account in personalizing care. It is conceivable that GST, with its emphasis on sharing feelings and connecting, is less suited for introverted people, which perhaps do better with IST. Autism might also be a barrier to fully participate in social group processes in GST. As a core feature of autism is dysfunction in theory of mind (i.e. understanding emotions, intentions, and other mental processes in other people), social group processes might be especially difficult to follow for participants with autistic features. Regarding trauma, for those who suffered severe levels of childhood abuse and neglect IST might be better suited than GST as there is more time for (individually tailored) extended trauma processing in IST. Finally, sleep problems are increasingly recognized as a factor contributing to maintenance of psychopathology and interfering with psychological treatment [23]. As there is more attention to individual problems such as sleep problems in IST than in GST, IST may have a better effect for those suffering from poor quality of sleep. Identifying predictors of treatment effect could help support clients and clinicians in their decision process as to which treatment might be selected for a given patient.

Despite its wide application in clinical practice, no direct evidence for the (cost)-effectiveness of GST for Cluster-C PDs exists. Before the implementation of this non-validated treatment is further disseminated, it is imperative to investigate the (cost-) effectiveness of GST for Cluster-C PDs. Common practice for assessing this for a new treatment is comparing it to the treatment as usual (TAU) to see whether further implementation is warranted. As GST is hypothesized to be a more cost-effective version of IST, a direct comparison between GST-IST is also desirable. By taking into account several predictors of treatment effect, a more detailed comparison can be made between the 3 therapies in order to gain insight into which treatment works best for whom.

### This study

This paper describes the QUEST-CLC study (QUalitative and Effectiveness study to Select optimal Treatment for CLuster-C PD), a multicenter RCT comparing GST, IST, and TAU for Cluster-C PDs. The main objective of the study is to compare the relative (cost-) effectiveness of these three treatments. Based on previous research, superiority of GST to TAU is expected regarding PD symptom reduction. How GST compares to IST in (cost-) effectiveness is yet unclear. Although in GST larger numbers can be treated in less therapist time than with IST, it is not yet clear how this relates to respective treatment effects. It is hypothesized that GST does not perform worse than IST regarding symptom reduction. As the few previous studies on treatment for Cluster-C PDs that do exist are generally limited to symptom reduction, measures of quality of life, social, work, and other functioning are also included. Furthermore, this study intends to identify predictors and moderators that are relevant for optimal treatment allocation. With such moderators a tool for treatment recommendation will be created. The tool is based on an empirically derived subset of several variables hypothesized to correlate with therapy outcome (i.e., diagnoses, age, gender, ethnic background, socio-economic status, educational level; introversion, autistic traits, sleep problems, severity of childhood traumas).

In addition, two sub studies will be carried out. First, the treatment integrity of GST and IST will be evaluated and compared with TAU. Second, a qualitative study will be performed in which patients and therapists are interviewed. The aim is to obtain a thorough understanding of how the different therapies helped patients recover, what elements are pivotal to lead a fulfilling life, and to explore experiences for patients in the ST-arms, in order to improve ST for Cluster-C patients. Moreover, ST-therapists’ perspectives about treatment will be used to further improve treatment protocols (as was done in the pilot study [24, 25]).

## Method

### Design

The study concerns a multicenter RCT with 3 treatment arms with unequal sizes GST (planned *n*=162), individual ST (IST, planned *n*=108), and TAU (planned *n*=108). Ten treatment sites in the Netherlands participate in this trial: Emergis, GGZ Oost-Brabant Helmond, Oss and Boxmeer, IPGGZ Veendam and Groningen, PsyQ Amsterdam, Rotterdam, Zaandam, and Utrecht (see Appendix B for organisational structure). This study has been approved by the psychology department’s Ethics Committee of the University of Amsterdam. This trial is registered at the Netherlands Trial Register; NL9209 and complies with the World Health Organization Trial Registration Data Set. Requests for modifications to the study protocol (e.g., adding questionnaires, altering study procedures) will be formally submitted to the Ethics Committee of the University of Amsterdam for approval.

### Recruitment

**Fig. 1:**
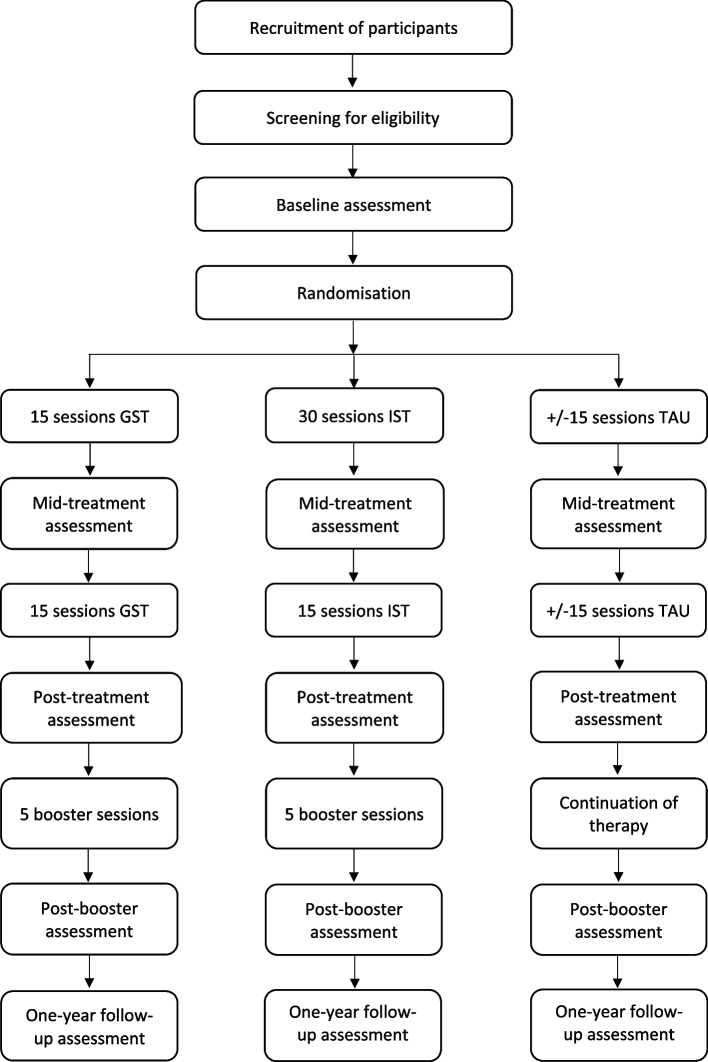
Flow chart of the study design

Participants are recruited from the regular influx of patients at the participating sites. Patients will be informed about study purposes and invited to participate in this study when diagnosed with Cluster-C PD or when Cluster-C PD is presumed. Informed consent (see Appendix A) will be obtained by a research assistant or clinician before the start of the baseline measurements. We strive for diversity and high representativeness in the sample by recruiting from the regular instream of patients, minimal in/exclusion criteria, and verifying that participants are not excluded for other reasons than listed in the exclusion criteria. Figure 1 provides a schematic overview of the patient flow starting with recruitment.

To ensure an adequate influx of participants in order to obtain the intended sample sizes, site-coordinators regularly update the central research team on the status of (and potential problems with) inclusion. Meetings are scheduled with sites facing inclusion problems so that tailored solutions can be implemented. Additional actions that are being undertaken to boost inclusion: recruiting a potential 11^th^ treatment site; recruiting more than the intended 42 patients at sites where inclusion fares well; increasing awareness of the study among prospective patients and referring clinicians by a website; organising a webinar aimed at educating referring general practitioners and clinicians on how to recognize Cluster-C PD and where to refer them.

### Patients

A total of 378 individuals with a primary Cluster-C PD diagnosis are planned to be recruited for this study. All diagnoses are determined by well-trained clinicians working in a multidisciplinary team. Inclusion criteria consist of (1) having received a primary diagnosis of APD, DPD, or OCPD based on the SCID-5-P [26]; F(2) being at least 18 years of age; (3) having sufficient Dutch language skills to participate in assessments and treatment; (4) being available and motivated to attend therapy for a period of one year regardless of offered treatment type (GST, IST or TAU). Additionally, participants are informed that they are not allowed to follow other psychological treatments in parallel to treatment received within the context of the study. Individuals are not eligible to participate if there is acute suicidality as determined by a clinician, or if they received a form of schema therapy in the last year.

### Sample size

Sample size calculations were based on symptom reduction effect sizes demonstrated in previous research on GST, TAU, and IST. In our pilot study, a 1-year pre-post effect size of d = 1.24 was found for GST on the primary outcome (severity of the primary PD). There is no data yet of TAU on this index. However, Bamelis and colleagues [12] found a difference between ST and TAU in recovery from PD-diagnosis of OR = 4.073 (81% (ST) vs 52% (TAU)). This is equivalent to an effect size d = .77. Secondly, in a recent multicenter trial, a difference between GST and TAU in reduction of BPD-severity of d = .73 was found [27]. Thirdly, the Svartberg et al. [28] RCT found a 1-year pre-post effect size of psychodynamic treatment, which is one of our TAU treatments, of d = .73 on an index of PD-severity. Taken together, it seems reasonable to assume a (large) pre-post effect size of TAU on PD-severity of d = .8, and also to assume a difference of d = .44 (1.24 – .8) between GST and TAU. Also note that this is a smaller effect size than found between ST and TAU in the Bamelis RCT [12] and between GST and TAU as treatments for Borderline PD [27].

The power analysis is based on 9 sites, despite 10 sites intent to participate, because sites might withdraw or not reach the intended N. For feasibility purposes a larger N is intended for the GST condition, as more participants can be treated at the same time in a group. With minimal N’s of 162 (GST), 108 (IST) and 108 (TAU) power of pairwise comparisons GST-IST and GST-TAU is 90% at an adjusted p-level of .025 to detect an effect size of d = .44 at post-test (or follow-up). For the GST-IST comparison, we don’t have expectations about the direction of a possible difference, but the power is sufficient to detect a medium effect size. One potential issue is treatment dropout. For pairwise comparisons of GST with the other two arms power is 80% to detect an RR = 2.5 at adjusted p = .025, for instance when dropout is 25% from GST vs. 10% from IST). (G)LMM analysis for repeated measures will be used to account for correlations introduced due to grouping (at multiple levels) and compensate for potential loss of power due to treatment drop-out. This type of analysis usually has greater power compared to non-multilevel/non-repeated measures analyses, however an actual power calculation for multilevel analysis with repeated measures and multiple sites is very difficult if not impossible because of the many random effects of the to-be-estimated model.

### Randomization

Randomization of the participants is conducted by an independent researcher after completion of the baseline assessment. Computerized covariate adaptive randomization is used, considering the primary diagnosis and gender. Six cohorts per site are planned, randomized approximately every 10 working weeks. It is possible that sites where inclusion runs smoothly will end up doing more cohorts relative to sites where inclusion falls behind. To guarantee balanced conditions over time, the aim is to randomize cohorts of 7 participants at a time per site resulting in GST (n=3), IST (n=2) and TAU (n=2). As participant influx may vary from time to time, occasionally less or more than 7 participants may be randomized. The distribution over the 3 conditions then will depend on 1) the capacity at the site in question, or 2) the need to allocate more participants to 1 particular condition in order to maintain the overall 3:2:2 distribution. At least 3 participants will be randomized per cohort as to ensure a chance for each participant to end up in one of the 3 conditions. The assigned intervention will be revealed to the patient in the first treatment session by the therapists.

### Assessments

Research assistants conduct semi structured interviews with participants to monitor severity of PD, clinical symptoms, and general functioning. Self-report questionnaires are administered on a PC on site. A distinction is made between tasks performed by a non-blind research assistant and a blind research assistant, the latter of which are kept blind to a participant's assigned treatment condition at all times. Both research assistants are allowed to perform all assessments before randomization, whereas after randomization only the blind research assistants are allowed to conduct the assessments. This is done to prevent any preferential bias a research assistant might have towards one of the treatments influencing how the interviews are conducted and interpreted. After randomization one subsection of the cost interview is assessed by the non-blind research assistant, as these questions relate to the participant's treatment. Audio recording of the interviews are made to assess the inter-rater reliability. Research assistants are instructed and trained by the two QUEST-CLC junior researchers (IG, A-SV).

In total five assessments take place: at baseline (week 0), mid-treatment (week 17), post-treatment (week 34), poster booster (week 51) and a follow-up 2 years after baseline. For the GST-arm this corresponds to a mid-treatment after 15 sessions, post-treatment after another 15 sessions, post-booster after 4 booster sessions and follow-up a year after termination of the therapy. IST follows the same pattern of GST, except that this arm starts with sessions twice a week, resulting in a mid-treatment after 30 sessions. For the TAU-arm the assessments are planned at the same time points (i.e., weeks), but note that these times might relate to different phases of the treatment than with GST/IST. For an overview of the primary and secondary outcome measures per assessment see Table 1. When more than 2.5 months have passed between a baseline assessment and start of therapy, an extra pre-treatment assessment is conducted in order to have a more reliable baseline measurement. For those patients in need of a pre-treatment assessment, the period between baseline and pre-treatment can be used as a proxy for a waitlist condition in later analyses. If patients decide to stop treatment, we will first try to motivate them to continue in the assessments and otherwise try to plan an exit-assessment starting with the primary outcome measures. There is a small financial renumeration of 40 euro per patients when they have completed all assessments.

**Table 1:** Overview of measures and assessment times

	Screening	Baseline	Pre-treatment	Mid-treatment	Post-treatment	Post-Booster	1 year Follow-up
SCID-5-S, SCID-5-P	•						
Screenings interview	•						
Biographical interview		•					
Demographics		•					
Severity index: AVPDSI/DPDSI/OCPDSI		•	•	•	•	•	•
Cost interview		•	•	•	•	•	•
PDBQ-69		•	•	•	•	•	•
RSES		•	•	•	•	•	•
MSGO		•	•	•	•	•	•
WHODAS 2.0		•	•	•	•	•	•
BSI		•	•	•	•	•	•
EQ-5D-5L & MHQoL-7D		•	•	•	•	•	•
Happiness Question		•	•	•	•	•	•
SMI-2		•	•	•	•	•	•
YSQ-SR		•	•	•	•	•	•
SLEEP-50		•	•	•	•	•	•
Introversion inventory		•	•	•	•	•	•
AQ-10		•					
CTQ-SF		•					
NLV		•					

*AVPDSI* Avoidant Personality Disorder Severity Index, *AQ-10* Autism Spectrum Quotient, 10 items, *BSI* Brief Symptom Inventory, *CTQ-SF* Childhood Trauma Questionnaire, *DPDSI* Dependent Personality Disorder Severity Index, *EQ-5D-5L* European Quality of Life, *MHQoL-7D* Mental Health Quality of Life seven dimensional questionnaire, *MSGO* Miskimins Self-Goal-Other Discrepancy, *NLV* National Adults Reading Test, *OCPDSI* Obsessive Compulsive Personality Disorder Severity Index, *PDBQ* Personality Disorder Belief Questionnaire, *RSES* Rosenberg’s Self-Esteem Scale, *SCID* Structured Clinical Interview for the DSM, *SMI* Schema Mode Inventory, *YSQ-SR* Young Schema Questionnaire, *WHODAS 2.0* WHO Disability Assessment Schedule

### Treatments

Schema therapy (ST) was developed by Young [13, 14] with the aim to improve interventions for individuals with (severe) personality disorders. ST integrates techniques and concepts from cognitive-behavioural, attachment and experiential therapies into an integrative model. Central to the ST model are the aspects of early maladaptive schema (EMSs) and schema modes. EMSs refer to relatively stable and broad character traits that children might develop in environments that failed to meet their core emotional needs (e.g., the need for safety, stability, acceptance, autonomy, etc.). EMSs are thought to endure throughout one’s life and when activated provoke intense negative emotions. Schema modes are defined as momentary states of mind which control the individual’s thinking, feeling, and acting at that moment. Contrary to EMSs, schema modes do not have a stable, trait-like character but can change continually. Through the use of the therapeutic relationship and experiential, cognitive and behavioural strategies, schema therapy aims to reduce maladaptive and strengthen functional schema and schema modes. In ST, the therapeutic relationship is conceptualised as limited reparenting, in which the therapist partly meets unmet needs within healthy therapeutic boundaries. In a directive and personal matter, the ST-therapist tries to offer a safe attachment, stimulates the patient to experiment with playfulness and sets boundaries.

#### Group schema therapy

GST consists of a one-year protocol [29] based on the Farrell & Shaw model [30] which was successfully piloted in 138 Cluster-C PD patients (see also [31] for preceding version of the protocol). In short, GST consists of 30 semi-closed group sessions in groups of 5-9 participants. GST is led by two therapists, which makes it possible to focus on both the individual and the group as a whole and, by means of limited reparenting, to meet underlying basic needs. Every 10 sessions participants who completed 30 GST sessions leave the group; the next session new participants enter the group, usually three. Prior to the group sessions, patients will receive two individual sessions of 1 hour to get acquainted with their therapists, explain GST, and identify the patient’s schema modes. In these sessions, the case conceptualisation is also drawn up based on the modes, schemas, EMS’s, coping and complaints. Next, patients participate in 30 weekly group sessions of 1.5 hour. The 30 group sessions are divided into three phases (covering approximately 10 weeks each) and are structured according to recurring elements. These elements include 1) welcoming group members; 2) attention and/or imagination exercise to here-and -now and to learn to cope with dysregulation; 3) announcements and reevaluation of last session 4) discussing homework; 5) addressing a new topic (e.g., a mode, techniques, or psychoeducation); 6) an experiential exercise; 7) reflection on the session; 8) review homework for next session; 9) wrap up the session with free-child exercise.

The three overarching phases denote the change each participant is hoped to make over the 30 sessions individually. Within the first phase patients work towards mode awareness and recognition of modes in everyday life. The second phase focuses on mode awareness and mode regulation through various techniques that focus on the patients’ behavior and experience as well as interpersonal techniques such as empathic confrontation. The last phase is aimed at maintaining behavioral change outside of therapy. Following these 30 sessions, four monthly booster sessions of 1 hour in small groups are offered to maintain and deepen the progress made. In addition, each participant receives max 300 minutes of individual ST at the patient’s or therapist’s request to discuss issues that may have been less suitable for the group or in times of crisis.

#### Individual schema therapy

IST intervention consists of 50 therapy sessions of 45 minutes. Therapy is provided two times a week for the first 30 sessions followed by 15 weekly sessions. The first five sessions are used for taking the life history, including adverse childhood experiences, listing problems and goals the patients aim to reach, case conceptualisation, and education in the model. During the next sessions that take place twice a week, at least once a week imagery rescripting on childhood memories related to the present PD-problems is performed. When the 15 weekly sessions start, attention shifts to present functioning and behavioural pattern breaking. In addition, five booster sessions with decreasing frequency are offered in the remaining months of the year. The IST protocol is described in detail in [15].

#### Treatment as usual

For TAU, usual procedures at the site will be followed in which the optimal treatment for the specific patient is chosen by experienced clinicians. No restrictions except for schema therapy with experiential elements are made for the TAU-condition. In this way, TAU mimics usual practice. Offered treatments for this study include Affect Phobia Therapy (AFT; individual or group), Cognitive Behavioural Therapy (CGT; part- or fulltime), Psychodynamic psychotherapy (individual or group), Guideline-Informed Treatment for Personality Disorders (GITPD), Short-term Schema Cognitive–Behavioural Group Therapy (SCBT-g [32];), or treatment by a psychiatric nurse. Although SCBT-g is sometimes viewed as a form of ST, we deemed it sufficiently different from ST delivered in the GST protocol because of its predominant use of cognitive techniques and its focus on the present. Importantly, experiential techniques and the specific use of the groups dynamics characteristic of GST are not part of the SCBT-g protocol.

### Drop-out, deviation from the treatment protocol, and follow-up

Patients are free to leave the study at any given time for any reason. However, when a patient refuses to participate in the assessments, the experimental treatment condition (i.e., IST and GST) will no longer be offered due to limited capacity of ST-therapists on the participating sites. Therapist can (in consultation with the researchers) decide to remove patients from treatment (i.e., pushout) in serious cases (e.g. when a client repeatedly fails to show up for treatment or seriously impedes the treatment process for fellow group members). Sites are responsible to provide a suitable alternative in case of drop- or pushout. Reasons for early termination of the treatment will be closely monitored and registered by the researchers in order to report these reasons in future publications. Deviation from the treatment length according to the ST-protocols is allowed when patients show sufficient progress in earlier phases (e.g., patients can independently apply advanced techniques by themselves). In this case the therapist is free to skip sessions in the protocol and or end treatment early.

Following both ST-protocols, we advise patients not to start a new treatment in the follow-up period and to apply the learned techniques on their own. Of course, we do not prohibit anyone from seeking help during this period. For the TAU condition this recommendation often does not apply, as therapy might not be finished yet. After having completed the follow-up assessment, the former therapist discusses the results with the patient as this was done during active treatment. Together, the therapist and patient evaluate whether there is a need for further therapy. If needed the therapist helps the patient determine what kind of therapy is fitting and where the patient can go if this therapy is not available at the respective treatment site.

### Therapists, Training and Supervision

Schema therapists, individual as well as group, are required to have followed the basic ST 25-hour training and have completed at least 10 supervisions whilst continuing supervision. In addition, GST therapists are required to have followed a 4-day Farrell & Shaw GST training as well as a 1-day training in the GST-CLC protocol by two skilled trainers [29]. Every GST group is conducted by two schema therapists and has a designated stand-in therapist who can replace the standard therapists when they are absent. This stand-in therapist needs to adhere to most of the aforementioned requirements except for having followed the 4-day F&S GST training. Therapists for the IST are also required to be either a junior schema therapist registered at the Dutch schema therapy association (VST) or in training to become one with accompanying continued supervision and a minimum of 10 supervisions completed. Additionally, IST therapists need to follow a 1-day training by one of the current authors (AA) in the IST protocol. It is possible for a schema therapist to be a GST as well as IST therapist, if they meet requirements for both treatment conditions.

Both GST and IST therapists engage in weekly peer supervision sessions at their site. GST and IST therapists are allowed to have these together. For the GST condition, each institute will receive up to 10 supervision sessions of 50 minutes by one of the creators of the GST CL-C protocol during the course of treatment (on average about once every two to three months). For IST two-hour online supervision every 6 months is offered for therapists who want to discuss specific issues. Such supervision is available to all sites. For the TAU condition no requirements are formulated regarding the training or supervision of therapists, as the treatments in this condition vary greatly and this condition is meant to reflect practice as usual at the institute. Therapists’ experience and credentials are gathered by a questionnaire designed specifically for trial therapists.

### Evaluation of Clinical Effectiveness

#### Primary outcome measures

##### Cluster-C Personality Disorder Severity Indices

Primary outcome is severity during the last month of the primary PD. For each Cluster-C PD a severity interview will be administered, assessing frequency of different manifestations of the pertinent PD as defined by the DSM-5 (Avoidant Personality Disorder Severity Index (AVPDSI [33];); Dependent Personality Disorder Severity Index (DPDSI [34];); Obsessive Compulsive Personality Disorder Severity Index (OCPDSI [35];). The AVPDSI consists of 53 questions divided into 7 subsections reflecting the 7 PD criteria. For each question both avoidance and fear over the past month are rated on a scale from 0 (never) to 5 (every day). The average avoidance per subsection and the average fear per subsection is calculated and summed up to form a total average avoidance and a total average fear score. The DPDSI consists of 47 questions divided into 8 subsections reflecting the 8 PD criteria, scored on a scale of 0 (never) to 5 (every day) over the past month. At the end of each subsection 2 additional questions are asked regarding the overall burden and impact of the subsection symptoms on a scale of 1 (not at all) to 10 (unbearably much). This results in a total sum of the average symptom scores per subsection, an average burden score and an average impact score. The OCPDSI is scored similarly, with 51 questions divided into 8 subsections reflecting the 8 PD criteria, scored on a scale of 0 (never) to 5 (every day) over the past month. At the end of each subsection again 2 additional questions are asked regarding the overall burden and impact of the symptoms on a scale of 1 (not at all) to 10 (unbearably much). This results in a total sum of the average symptom scores per subsection, an average burden score and an average impact score. These interviews have excellent interrater agreement (ICC>.90) and internal consistency (Cronbach a > .90) and are sensitive to change. By standardizing the raw scores (dividing by the baseline SD) the scores on the three instruments are converted into one severity score.

#### Secondary outcome measures

##### Personality Disorder Beliefs

The Cluster-C PD subscales of the Personality Disorder Beliefs Questionnaire (PDBQ [36];) consist of 32 questions rated on a visual analogue scale (1-100). Thirteen questions reflect beliefs characteristic of the dependent PD, 10 reflect avoidant PD beliefs, and 9 reflect obsessive-compulsive PD beliefs. Three mean scores are calculated for these Cluster-C PD-specific beliefs. Internal consistencies as estimated by Cronbach’s alpha coefficients of the 3 subscales are all in the range of good to excellent (.85 – .95 [37];). The sum of the 3 subscales will be used.

##### Self-Esteem

Self-esteem is measured by the Rosenberg’s Self-Esteem Scale (RSES [38]; Dutch version: [39]). The questionnaire consists of 10 statements with a Likert scale of 4 possible answers ranging from 0 to 3 (“highly agree” - “highly disagree”). The sum score reflects one’s level of self-esteem. The Dutch RSES showed high internal consistency (α = .86) as well as high congruent validity [39].

##### Self-Ideal Discrepancy

The discrepancy between the ideal self and the perceived self is measured by the Miskimins Self-Goal Other Discrepancy Scale (MSGO [40];). The MSGO contains 15 items rated on a visual analogue scale, one side of the scale displaying a characteristic relating to the self (such as “happy”) and the other side displaying the opposite of this characteristic (such as “unhappy”). Each item is rated twice, once “as I am now” and once “as I would like to be”. The item score reflects the distance between the ideal-real ratings on each dimension (0-100), the total score is the average discrepancy. The MSGO has good reliability and validity [40].

##### General Psychopathology

The Brief Symptom Inventory (BSI) is a self-report instrument that measuring general psychopathology [41]. It consists of 53 items addressing nine primary symptom dimensions: somatic, cognitive, inter-personal sensitivity, depressive mood, anxiety, hostility, phobia, paranoia, and psychoticism. Answers are scores on a 5-point Likert Scale. The total score will be used. Internal consistency for the Dutch version is excellent (⍺ = .96) and the discriminant validity good [42]**.**

##### Functioning

Problems with general functioning are measured by the World Health Organisation Disability Assessment Schedule II (WHODAS 2.0 [43];. The WHODAS interview includes 36 items addressing six functional domains: cognition, mobility, self-care, getting along with people, life activities at work and home, and participation in society. Items can be answered on a 5-point Likert scale ranging from ‘no difficulties’ to ‘extreme difficulties ‘. Internal consistency is very high: Cronbach’s alpha varies from .91 to .97 between different domains. The WHODAS 2.0 has also proven to be sensitive to changes in functioning after therapies [44]. The total scale will be used.

##### Quality of Life (QoL)

To obtain a potentially more valid estimation of quality of life we use two self-report questionnaires: the EQ-5D-5L and the Mental Health Quality of Life Questionnaire (MHQoL-7D [45];. In contrast to the EQ-5D-5L, the MHQoL-7D has been developed specifically for mental health patients. Hence it focuses more on mental aspects of quality of life than the EQ-5D-5L, which is dominated by physical (somatic-medical) aspects of quality of life. However, the validation of the MHQoL-7D and the development of an algorithm for utilities (necessary for the economic analysis of our study) are not yet completed, therefore it is premature to replace the EQ-5D-5L with the MHQoL-7D. Both (very short) questionnaires will therefore be administered.

#### EQ-5D-5L

The EQ-5D-5L [46] contains five dimensions of health-related quality of life: mobility, self-care, daily activities, pain/discomfort, and depression/anxiety. Additionally, the EuroQol thermometer ranging from 0 to 100 provides a single index measure of the patient’s health status. The intraclass correlation of the EQ-5D-5L ranges from .73 to .84 for the summary index [47]. The total score of the five dimensions will be used as a measure of QoL.

#### MHQoL-7D

The MHQoL-7D [45] is a self-administered measure of QoL, specifically developed for individuals with subclinical and clinical mental health problems. It consists of two parts: the MHQoL-7D and the MHQoL-VAS. The MhQoL-7D addresses seven domains (self-image, independence, mood, relationships, daily activities, physical health, future) with four response levels ranging from very dissatisfied (score = 0) to very satisfied (score = 3). The total score can range from 0 to 21, with higher scores indicating higher QoL. In addition, the MHQoL-VAS measures the self-esteemed general psychological well-being on a scale ranging from 0 (worst imaginable psychological well-being) to 10 (best imaginable psychological well-being). The Dutch version of the MHQoL-7D has a good internal consistency reliability (Cronbach’s alpha = .85) and has shown to be able to distinguish between participants with and without clinical psychopathology [45]. In addition to the trial registration, we plan on using the total score as a secondary outcome measure.

##### Happiness Question

General happiness in the months prior to assessment is measured with a single item [48]. Answered are scored on a 7-point Likert Scale, with the following options: 1) completely unhappy, 2) very unhappy, 3) fairly unhappy, 4) neither happy nor unhappy, 5) fairly happy, 6) very happy, 7) completely happy. This single item has proven to have good convergent and divergent validity with relevant constructs and has a high test-retest reliability (r = .86 [48];).

##### Schemas

The Young Schema Questionnaire – Short Form (YSQ – SR [49];) is a self-report questionnaire aimed at measuring 18 Early Maladaptive Schemas (EMSs). It contains 90 items scored on a 6-point Likert scale (“not true at all”- “completely true”). Average item scores are calculated per schema subscale. The YSQ-SR has high overall internal validity (α = .94 - .96) and satisfactory to high internal consistency for the subscales (α = .72 - .94 [50];). The total score will be used as an index of EMSs.

##### Schema Modes

Schema Modes are measured with the Schema Mode Inventory-2 (SMI-2 [51];), which measures the extent to which 18 schema-modes are present in the period around time of administration. The SMI is a 192-item scale rated on a 6-point Likert scale (“never or almost never” – “always”). Average item scores are calculated per schema-mode subscale. The SMI-2 is only used for patients in the GST and IST conditions. The SMI-2 has shown good internal consistency [51]. An index of maladaptive schema modes and one of adaptive schema modes will be used.

##### Sleep Quality

The SLEEP-50 is a self-report instrument on sleep quality [52]. The questionnaire consists of 50 questions with 4 answer options (ranging from “not” to “a lot”) reflecting nine domains: Sleep Apnea, Insomnia, Narcolepsy, Restless Legs/PLMD, Circadian Rhythm Sleep Disorder, Sleepwalking, Nightmares, Factors Influencing Sleep, and Impact of Sleep complaints on daily functioning. The answers to these 50 questions are summed to form a total score of sleep quality (higher scores reflecting lower sleep quality). Additionally, a subjective grade is asked concerning sleep quality (1-10), as well as the time of going to sleep and waking up. The internal consistency for the entire scale is high (Cronbach alpha = .85). Test-retest reliability ranges from .65 - .89 [52].

##### Introversion

To assess introversion, the authors have developed a questionnaire due to lack of short and open-source instruments that focus on characteristics of introversion rather than the absence of the trait extraversion. The ‘Introversion Inventory’ [53] is based on the Big-5 Facet theory underlying the NEO-PI-R ([54] extraversion scale. The Introversion Inventory has 19 items with answering options ranging from 0 (completely incorrect) to 4 (completely correct). A (translated) example question is ‘In company I am reserved’. This new scale will be validated in this and adjacent studies and made available as an open-source instrument. Although introversion is hypothesized to be a predictor, this questionnaire was also added to the repeated measurements to see if changes occur over time due to influences of (group) therapy.

#### Predictors-moderators

##### Autism Characteristics

The Dutch Autism-Spectrum Quotient-10 (AQ-10 [55]; is used to measure autism characteristics. The AQ-10 consists of 10 items scores on a 4-point Likert scale, determining one’s difficulties with social skills, routine, imagination, detail-focus, and attention switching. The total score ranges from 0-10, with a higher score indicating more autistics traits. A score of 6 or higher is indicative for a full diagnostic assessment [56]. The internal consistency of the AQ-10’s total score is high (α = .85 [57];). The AQ-10 is able to distinguish between individuals with and without a clinical diagnosis of ASD [56].

##### Childhood Trauma

The Childhood Trauma Questionnaire – short form (CTQ-SF [58];) is used to assess the severity of childhood maltreatment. The short form consists of 25 items that are depicted to measure 5 subscales of childhood trauma: physical, sexual, and emotional abuse and physical and emotional neglect. Answers are measures on a 5-point scale ranging from 1 (never true) to 5 (very often true), resulting in a total score of the subscales between 25-125, with higher scores reflecting more childhood trauma. The total score of the subscales will be tested as predictor and moderator of treatment effects. A Dutch validation study showed that the CTQ-SF has a good internal consistency (Cronbach’s alpha > .87 for the separate scales) and showed a good convergent validity with scores on a semi-structured interview for childhood trauma [59].

##### Verbal Intelligence (NLV)

Verbal intelligence is assessed by the Dutch version of the National Adult Reading Test (NLV [60];). For this test a list of 50 words with an uncommon pronunciation is read out loud by the patient. For every correct pronunciation 2 points are awarded, for every unclear or questionable pronunciation 1 point is given, and for incorrect pronunciation no points are given. The total score consists of the aggregated points.

##### Demographics

A semi-structured demographical interview will be used to collect some general patient characteristics. The age, gender, marital status, sexual orientation, ethnic and cultural background, level of education, employment situation, previous psychological treatments, among other things, will be assessed.

##### Analyses

All analyses are planned to be carried out by SPSS or R statistical software and Excel (for Bootstraps).

Primary and secondary outcomes are analysed with Linear Mixed Models (LMM), with random effect of site. As data of group members are not independent because group members might influence each other, this dependency needs to be taken into account. With the semi-closed group format, groups change in composition every 10 sessions, and participants will share different amounts of time in sessions with group peers: from all group sessions to 10 group sessions. It is plausible to model covariances as a function of the amount of time (i.e., sessions) patients have spent together in a group. This can be achieved in a multilevel model with repeated measures by specifying a Toeplitz covariance structure to model this dependency on the group level. In case this Toeplitz structure turns out to be unsuitable to model the dependency, another (less complex) structure will be specified. For the repeated part at the individual level, the best fitting covariance structure will be chosen (e.g., AR1, ARMA11, Unstructured). If residuals deviate substantially from normal distribution, Generalized LMM (GLMM) will be used with an appropriate distribution (e.g., negative binomial or gamma in case of skewed distributions). All analyses are conducted on the basis of intention-to-treat and will be controlled for initial symptom severity. Dropout from treatment will be analysed with GLMM survival analysis with site as random effect. To take into account participants’ potential influence on each other in group treatment, again a Toeplitz covariance structure will be specified to model this dependency unless a different structure turns out to be more suitable. The three arms will be mutually compared, with a primary interest in the GST-TAU and the GST-IST comparisons. Although the IST-TAU difference is not part of the main research questions, this difference will be analysed exploratively. Tests of predictors and moderators (sleep quality, introversion, autism traits, childhood trauma, verbal intelligence, demographic characteristics) will be done by similar (G)LMM analyses, with the predictor, predictor x time, predictor x treatment, and predictor x time x treatment interactions added to the model.

To derive a tool that informs future patients and therapists what treatment is likely to be most efficacious for the specific patient (personalized care), we currently plan to use the Personalized Advantage Index (PAI) procedure developed by DeRubeis [61]. The LMM model of the outcome analysis is the starting point. Then, potential predictors and moderators are derived by suitable predictive machine learning models. Two methods for model selection, best subset selection and Lasso regression, will be performed and validated using 10-fold cross validation, after which one of the two methods will be selected. The individual’s PAI and hence the advantage of optimal treatment allocation can be estimated with a leave-one-out procedure [61, 62]. However, the field of personalized treatment allocation is developing rapidly and other methods might turn out to be more accurate and robust than the PAI method. We will therefore use a method that is optimal given the state of affairs at the end of data collection.

### Economic Evaluation

The economic evaluation will involve a combination of cost-effectiveness analysis (CEA) and cost-utility analysis (CUA). In a CEA, effects are presented in clinical outcomes (here the primary outcome is Cluster-C PD-severity decrease). The outcome for the CUA is Quality Adjusted Life Year’s (QALY’s), based on the EQ-5D-5L utility scores [46], and the MHQoL-7D [45] for a full description see above) primarily on Dutch utilities [63]. In the CUA, the Incremental cost-effectiveness ratio (ICER) will be expressed as incremental costs per QALY.

#### Cost measurement

For this study we have developed a cost interview especially designed for this group, based on existing questionnaires, such as the de Trimbos/iMTA questionnaire for Costs associated with Psychiatric Illness (TIC-P) [64]. Since PDs are known to interfere with all life aspects, this economic evaluation will be performed from a societal perspective, which implies that all relevant costs and outcomes are considered. Health costs covered intervention, healthcare, social work, emergency department, community health service, informal and alternative care. Costs outside the health care consist of comprised losses in productivity of (unpaid)work, study, and domestic activities up to two years, traveling costs, out-of-pocket costs (e.g., alcohol, tobacco, drugs, impulsive spending), and judicial costs. Costs are measured at baseline before random assignment to treatments, and every assessment for 2 years, using a 4-month recall period.

Total costs will be estimated using a bottom-up (or micro-costing) approach, where information on each element of service used is multiplied by an appropriate unit cost and summed to provide an overall total cost [65]. The valuation of healthcare, patient and family costs will be based on the updated Dutch manuals for cost analysis in healthcare research [66]. In brief, the manual recommends standardized cost prices for health care and shadow prices for informal care (meaning a standard cost price based on general hourly wages). Costs of transport will be calculated as the mean distance per destination multiplied by standard cost prices. Costs of medication will be calculated using prices based on Daily Defined Dosage (DDD) taken from the healthcare institute of the Netherlands^1^ indicating the mean medication usage per adult a day inclusive claw back (by the government-imposed discount for patients, paid by the pharmacy). Productivity losses will be calculated by means of the friction cost method, based on a mean added value of the Dutch working population. The friction costs method considers production losses confined to the period needed (usually 90 days) to replace a sick employee. In case of uncertainty, we will use a conservative estimation (i.e., the lowest cost price). Cost prices will be expressed in 2021 euros. If necessary, existing cost-prices will be updated to 2021 using the consumer price index (CPI). Interrater agreement of the cost interview will be assessed by blinded double coding.

### Cost-Effectiveness and Cost-Utility Analyses

A baseline analysis will be performed to examine the comparability of groups at baseline for both costs and outcomes. We aim to use the multiple imputation method to handle missing values at item level using the individual mean score [67]. However, we will monitor recent developments in the rapidly advancing field of cost-effectives studies to decide on the most fitting technique when time is due. If necessary, methods are applied to control for differences in baseline [68]. The ICER will be determined based on incremental costs and effects of group-ST compared to TAU. The cost-effectiveness ratio will be stated in terms of costs per outcome rate, the cost-utility ratio will focus on the net cost per QALY gained. The ICER will be calculated as follows. ICER = (Ci – Cc) / (Ei – Ec), where Ci is the annual total cost of the GST, Cc is the annual total cost of TAU (resp. IST), Ei is the effect at 2-years follow-up for GST and Ec is the effect at 2-years follow-up for TAU (resp. IST). The three arms will be mutually compared, with a primary interest in the GST-TAU and the GST-IST comparisons. However, the IST-TAU difference will also be tested. The robustness of the ICER will be checked by non-parametric bootstrapping. Bootstrap simulations will also be conducted to quantify the uncertainty around the ICER, yielding information about the joint distribution of cost and effect differences. The bootstrapped cost-effectiveness ratios will be subsequently plotted in a cost-effectiveness plane, in which the vertical line reflects the difference in costs and the horizontal line reflects the difference in effectiveness. The choice of treatment depends on the maximum amount of money that society is prepared to pay for a gain in effectiveness, which is called the ceiling ratio. Therefore, the bootstrapped ICERs will also be depicted in a cost-effectiveness acceptability curve showing the probability that group-ST is cost-effective using a range of ceiling ratios.

Additionally, to demonstrate the robustness of our base-case findings a multi-way sensitivity analysis will be performed. In the sensitivity analysis uncertain factors of assumptions in the base case analysis are recalculated to assess whether the assumptions have influenced the incremental cost-effectiveness ratio (ICER), for example by varying cost-prices and volumes between minimum and maximum [69]. For the primary research question, GST is compared to TAU. For the secondary research question, GST is compared to IST. In addition, IST will be compared to TAU.

### Qualitative study

The qualitative sub study has a threefold objective; 1) Obtain thorough understanding into whether and how all treatments helped patients recover and which elements are pivotal to lead a fulfilling life; 2) Explore the experiences for patients and their loved ones in the ST-arms, and 3) Examine perspectives of ST-therapists about the treatments protocols so that they can be further improved (as was done in [12, 25].

In-depth semi-structured interviews of maximally one hour will be held among 15 patients per arm. These interviews will be composed using a thematic analytic approach [70], combining a top-down (i.e., themes are pre-formulated) and bottom-up approach (new themes arising from the interviews). A topic list for this qualitative study is formulated in co-creation with the expert panel of the study board. By letting participants comment on summaries of their interviews, a member check is done that we adequately understood them [71]. Furthermore, the interview will first be tested in 2-3 participants per arm, revised and then used in the subsequent interviews [72]. Sampling of participants will be done based on diversity [73] instead of statistical representativeness. In the proposed purposeful sampling, we aim to have diversity in primary diagnosis, site, gender, age, ethnic background, success or failure of treatment, and completion or premature termination of treatment. In addition, we will ask 20 carers and family members of patients who participated in this trial about how they experienced the therapy of their loved one. Finally, we plan on interviewing 10 therapists in the ST-arms about their experiences with the treatment protocols to further improve these.

### Additional sub studies

#### Treatment Integrity

To assess the treatment integrity of the GST and IST conditions, trained independent raters will score a random sample of video tapes of treatment sessions of each arm using already available adherence-checklists [74]. Per site at least 30 videos will be rated per treatment arm. In particular we will compare GST to the groups in the TAU arm, specifically Short-term Schema Cognitive–Behavioural Group Therapy (SCBT-g [33];), as there is theoretical overlap between the two protocols, but little overlap in techniques and handling of the group dynamics. The two therapies will be compared on each respective treatment integrity instrument to assess their similarities and differences in therapy-specific elements. Additionally, other TAU group therapies (such as GIT-PD) will also be assessed on these instruments. Finally, all GST, IST and TAU video’s will be assessed on general therapeutic characteristics such as explicit directiveness and empathy scale.

#### Validation of the Introversion Inventory

The authors developed a questionnaire to assess introversion due to lack of short and open-source instruments that focus on characteristics of introversion rather than the absence of the trait extraversion. The Introversion Inventory will be validated in the current sample as well in a non-clinical PD sample.

### Monitoring

#### Data collection and storage

The data will be collected in collaboration with research assistants at the participating mental health institutes. To monitor and support data collection, a web tool named ‘Lotus’ will be used. Lotus is developed by the University of Amsterdam and monitors the trajectory of patients, reminding the research assistants and junior researchers of an upcoming assessment. Lotus is linked to a secure online survey software called Qualtrics, where anonymised data is temporarily stored on a secure server of the University of Amsterdam. Research assistants register new patients in Lotus, where they receive a unique identifier to ensure data collection to be as anonymous as possible. A list of identifiers linked to personal information is only accessible for the research assistants and site coordinator of a specific site. Institutional network research storage will be used to storage and backup the data. Clear arrangements have been made about ownership of the data and responsibility in the research process, which is all disclosed in a collaboration agreement. No periodic inspection of the accumulating outcome data is needed; hence no DMC exists for this trial. Data collection will continue until the intended N has been reached, therefore no interim analyses are needed to inform a decision on the termination of the trial.

#### Safety

We don’t expect increased risks for patients participating in the different treatments given the results of earlier studies (see background). To reduce risks of any harms, we explicitly use acute suicidality as exclusion criterion. In case of any serious adverse events (SAE, i.e., any adverse event that results in death or is life-threatening, requires hospitalisation or results in significant disability), the junior researchers will be notified within 24 hours of notice. In every SAE, we will investigate the relatedness to the intervention and will report the SAE to the ethical committee of the University of Amsterdam. Because there are low rates of SAE’s expected, no specific hypotheses were formulated to incorporate in the analysis.

## Discussion

In this article the study design of a multicenter RCT into the (cost-)effectiveness of GST relative to IST and TAU for Cluster-C PDs is described. This study design will contribute to the urgent need to create treatment guidelines for individuals with Cluster-C PD and will broaden our understanding of which aspects help Cluster-C PD patients to recover and live a fulfilling life.

The main objective of this study is to assess the evidence of GST for Cluster-C PDs over IST and TAU. Group-treatment has potentially unique therapeutic advantages such as peer-support, vicarious learning, and a safe environment to practice new behaviors. Additionally, GST is already a popular treatment in clinical practice in the Netherlands and many other countries due to (assumed) advantages such as being able to treat more patients in a shorter period of time, offering a solution to the long waitlists for PD-treatment. If GST is indeed found superior to IST or TAU, this would greatly reduce pressure on the Dutch mental healthcare system. The fact that clear guidelines and protocols for GST already exist facilitates training and implementation.

This trial includes a comprehensive economic evaluation where the additional costs and additional outcomes of GST compared to IST and TAU are estimated. The gold standard for assessing the cost-effectiveness of a new treatment is comparing it to TAU. For policy makers, the comparison of GST to TAU is pivotal in the decision-making process of implementing a new treatment in clinical practice. In this specific case, a direct comparison of GST to IST is also important. First, only a direct comparison will tell us to what degree GST is cost-effective compared to IST. Admittedly, the direct delivery costs per client of GST are estimated to be 50% of that of IST. However, we don’t know how other (healthcare) costs compare between GST and IST, and how the clinical effects compare. Therefore, a direct investigation of the comparative cost-effectiveness of GST and IST is needed.

In addition to determining the relative (cost)-effectiveness of the three treatments, this study aims to elucidate which treatment works best for whom. It is likely that some clients are better off with GST and others with IST or TAU. The development of a treatment allocation tool would greatly support clients and clinicians in their decision process by giving an indication of the improvement that can be expected per treatment based on the client's specific profile. Matching treatment to client has the potential to reduce societal costs, improve treatment outcomes, reduce drop-out and combat demoralization in clients with a long treatment history.

Finally, a qualitative study will give insight into *how* therapies helped patients recover and explore what elements are essential for clients to lead a meaningful life. The use of a semi-structured interview makes it possible to identify these underlying processes in a more personalized manner and can help explore new and unidentified visions/ideas in recovery processes relevant for this population. Moreover, these insights can be used to further improve the treatment protocol for ST. ST-therapists’ perspective and experiences will also be explored to further improve the protocol(s).

The QUEST-CLC study design has several methodological strengths: First, a highly diverse and representative sample will be included by minimizing in- and exclusion criteria, enhancing the generalizability of the results into real life practice. Second, all questionnaires and semi-structured interviews represent scientifically sound measures which will be assessed by trained research assistants who are blind to treatment conditions in order to control for interviewer bias. Third, the repeated measurements give the opportunity to closely monitor changes over time and detect possible working mechanisms within treatment. Additionally, the long-term follow-up assessment will give insight in patients’ continuance when treatment is ended. This is particularly interesting given that the pilot study of this GST protocol showed that the significant decrease in symptoms continued after termination of treatment.

Naturally, a clinical, multicenter RCT involves complex decisions to ensure the quality and feasibility of the trial. Here we outline some of these key considerations and challenges. Firstly, as TAU will be the primary treatment offered to the individual patient at each participating site, TAU reflects the current optimal clinical practice in the Netherlands. The variety of TAU types over the 10 sites spread over the Netherlands is meant to be representative of the current usual treatment of Cluster-C PDs in clinical practice. A downside to not formulating guidelines for the TAU is that little control can be executed over treatment-specific variables that are more fixedly regulated in the IST and GST conditions (e.g., frequency and intensity of treatment, therapist training and supervision). Nevertheless, protocolized treatments like Affect Phobia Therapy and SCBT-g are currently quite common in Dutch mental health care and are usually given by trained therapists. To document the TAU provided, questionnaires are administered to register treatment as well as therapist characteristics that can be entered into later analysis.

Next to the advantages of conducting research in multiple mental health centers regarding generalizability, it also introduces potential bias of characteristics related to sites rather than treatments (different location, patient demographics, therapists, etc.). These non-treatment specific variables can differentially influence treatment outcomes and can complicate the interpretation of the results (hence reducing generalizability). We therefore will use multilevel analysis that takes this structure of the data into account. Multilevel analysis allows for modelling variance at the site-level and differentiating treatment-specific from site-specific influences.

Another point of attention for multicenter studies is to organize and coordinate all participating sites in a clear manner. Maintaining the quality of the study protocol over 10 sites is a demanding task which requires considerable work and effort. The two junior researchers are the central figures of the study and keep in close contact with the local research assistants and coordinators of all sites. They oversee the daily coordination of the study, prepare the meetings, monitor the progress of the study and its legal, ethical and ICT aspects. Monthly meetings are held with all site coordinators to discuss the implementation of the study and to address potential difficulties. In addition, every six months the study board (site coordinators, experience experts, project leaders, the statistician and economical expert) gather to discuss developing and setting up new components to the study protocol. Finally, we (i.e., project leaders and junior researchers, independent of the sponsor) plan on auditing every site once during the inclusion period. After that, auditing will be conducted using a risk-based approach at sites who show higher rates of dropout, lower inclusions, less accuracy in assessments etc.

As previously stated, this trial consists of an extensive economic evaluation. To express the effects of the intervention we have chosen to include a cost-effectiveness and a cost-utility analysis to explore both PD-specific improvements as QALY’s. Moreover, we have chosen to incorporate two measurements to explore quality of life: the commonly used EQ-5D and the relatively new MHQoL-7D. While the EQ-5D is dominated by physical (somatic-medical) aspects of quality of life, the MHQoL-7D focusses more on mental aspects of quality of life (e.g., mood, self-image, future perspectives etc.) making it especially relevant for this population. Incorporating both questionnaires will give us an interesting opportunity to compare these measures for their suitability for a mental health population. For the economic evaluation we have chosen a time horizon of two years covering the one-year treatment and a follow-up period of one year. Although ideally one would want to choose a time horizon to cover all long-term costs and consequences (i.e., up to a lifetime), this relatively lengthy period gives the opportunity to explore the most essential period of change.

Another challenge in economic evaluations is to properly identify all relevant consequences and costs for specific populations. Because personality disorders are known to affect all life aspects a societal perspective has been chosen to be able to assess the costs in their entirety. In the development of the cost interview, we especially focused on some patient-specific characteristics, for example production losses at work, study, or domestic activities (due to possible avoidance or perfectionism), detailed questions of psychotropic medications (due to high comorbidity with mood- and anxiety disorders), and an extensive determination of health costs (because of possible higher somatization within this cluster). Nevertheless, we will not be able to determine all PD-associated costs such as problems in cooperating with colleagues, disrupting others in work, difficulties with fulfilling the role of parent [10]. In addition, it remains difficult to determine whether costs are tied to PD-related problems or whether people incur these costs anyway. Adding a control group when identifying costs could give a definitive answer on this.

For the current investigation, patients with an otherwise specified PD with predominantly Cluster-C traits were not included. There are various reasons for excluding these patients. First, these patients are less clearly defined than those with a specified Cl-C PD, which is problematic for generalizing findings. Second, research has documented that their level of impairment is considerably less than that of patients with specified PDs. Third, with our severity instruments, it would be difficult to track their progress in PD-severity. The three main outcome measures are developed for one of the three Cluster-C PDs specifically, hence it is not possible to reliably track progress for those with an otherwise specified PD with predominantly Cluster-C traits. Additionally, we are interested in differences between the 3 conceptually different Cluster-C PDs and want to see whether treatment recommendation is different depending on primary diagnosis.

In conclusion, this randomized controlled trial focuses on a clinical population that has so far been unjustly ignored in the scientific field, despite its high prevalence and severe consequences for the patients and society. Findings from this study will help determine future directions for the effective treatment of Cluster-C PDs regarding treatment type as well as matching patient to treatment. GST holds great promise as a (cost)-effective treatment for many, but probably not all Cluster-C PD patients as it can potentially offer a considerable improvement of quality and efficiency of health care.

## Supplementary Information

**Additional file 1.**

## Data Availability

Not applicable.

## Abbreviations

AFT: Affect Phobia Therapy
AVPDSI: Avoidant Personality Disorder Severity Index
AQ-10: Autism Spectrum Quotient, 10 items
BSI: Brief Symptom Inventory
CBT: Cognitive Behavioural Therapy
CTQ-SF: Childhood Trauma Questionnaire
CEA: Cost-Effectiveness Analysis
CUA: Cost-Utility Analysis
DPDSI: Dependent Personality Disorder Severity Index
EQ-5D-5L: European Quality of Life
GIT-PD: Guideline-Informed Treatment for Personality Disorders
GST: Group Schema Therapy
ICER: Incremental Cost-Effectiveness Ratio
IST: Individual Schema Therapy
MHQoL-7D: Mental Health Quality of Life Seven-Dimensional questionnaire
MSGO: Miskimins Self-Goal-Other Discrepancy
LMM: Linear Mixed Models
NLV: National Adult Reading Test
OCPDSI: Obsessive Compulsive Personality Disorder Severity Index
PAI: Personalized Advantage Index
PD: Personality Disorder
PDBQ: Personality Disorder Belief Questionnaire
RSES: Rosenberg’s Self-Esteem Scale
SAE: Severe Adverse Event
SCBT-g: Short-term Schema Cognitive–Behavioural Group Therapy
SCID: Structured Clinical Interview for the DSM
SMI: Schema Mode Inventory
ST: Schema Therapy
TAU: Treatment as Usual
YSQ-SR: Young Schema Questionnaire
WHODAS 2.0: WHO Disability Assessment Schedule
QALY: Quality Adjusted Life Years
QoL: Quality of Life

## Footnotes

1 https://www.medicijnkosten.nl/

## References

[1] Volkert J, Gablonski T-C, Rabung S. Prevalence of personality disorders in the general adult population in Western countries: systematic review and meta-analysis. Br J Psychiatry. 2018;213:709–15. 30261937 10.1192/bjp.2018.202

[2] Winsper C, Bilgin A, Thompson A, Marwaha S, Chanen AM, Singh SP, et al. The prevalence of personality disorders in the community: a global systematic review and meta-analysis. Br J Psychiatry. 2020;216:69–78. 31298170 10.1192/bjp.2019.166

[3] Epidemiology TS. In: Widiger T, editor. The Oxford Handbook of Personality Disorders: Oxford University Press; 2012. p. 186–205.

[4] Hutsebaut J, Willemsen EMC, Van HL. Time for cluster C personality disorders: state of the art. Tijdschr Psychiatr. 2018;60:306–14. 29766478

[5] Chen H, Cohen P, Crawford TN, Kasen S, Johnson JG, Berenson K. Relative impact of young adult personality disorders on subsequent quality of life: Findings of a community-based longitudinal study. J Pers Disord. 2006;20:510–23. 17032162 10.1521/pedi.2006.20.5.510

[6] Coid J, Yang M, Tyrer P, Roberts A, Ullrich S. Prevalence and correlates of personality disorder in Great Britain. Br J Psychiatry. 2006;188:423–31. 16648528 10.1192/bjp.188.5.423

[7] Samuels J, Eaton WW, Bienvenu OJ, Brown CH, Costa PT, Nestadt G. Prevalence and correlates of personality disorders in a community sample. Br J Psychiatry. 2002;180:536–42. 12042233 10.1192/bjp.180.6.536

[8] Soeteman DI, Hakkaart-van Roijen L, Verheul R, Busschbach JJ. The economic burden of personality disorders in mental health care. Journal of Clinical Psychiatry, 69(2), 259. Journal of Clinical Psychiatry. 2008;69:259–65. 10.4088/jcp.v69n0212 18363454

[9] Olssøn I, Dahl AA. Avoidant personality problems—their association with somatic and mental health, lifestyle, and social network. A community-based study. Compr Psychiatry. 2012;53:813–21. 22146705 10.1016/j.comppsych.2011.10.007

[10] Bamelis LLM, Arntz A, Wetzelaer P, Verdoorn R, Evers SMAA. Economic evaluation of schema therapy and clarification-Ooriented psychotherapy for personality disorders. J Clin Psychiatry. 2015;76:e1432–40. 26579561 10.4088/JCP.14m09412

[11] Bachrach N, Arntz A. Group schema therapy for patients with cluster-C personality disorders: A case study on avoidant personality disorder. J Clin Psychol. 2021;77:1233–48. 33538340 10.1002/jclp.23118

[12] Bamelis LLM, Evers SMAA, Spinhoven P, Arntz A. Results of a multicenter randomized controlled trial of the clinical effectiveness of schema therapy for personality disorders. J Psychiatry. 2014;171:305–22. 10.1176/appi.ajp.2013.12040518 24322378

[13] Young JE, Klosko JS, Weishaar ME. Schema Therapy: a practitioner’s guide. New York: The Guilford; 2003.

[14] Young JE. Cognitive therapy for personality disorders: A schematic-focused approach. Sarasota: Professional Resource Exchange; 1990.

[15] Arntz A. Schema therapy for cluster-C personality disorders. In: Vreeswijk M, Broersen J, Nadort M, editors. The Wiley-Blackwell handbook of schema therapy: theory, research and practice. Chichester: Wiley-Blackwell; 2012. p. 397–414.

[16] Jacob GA, Arntz A. Schema therapy for personality disorders—A review. Int J Cogn Ther. 2013;6:171–85.

[17] Farrell JM, Shaw IA, Webber MA. A schema-focused approach to group psychotherapy for outpatients with borderline personality disorder: A randomized controlled trial. J Behav Ther Exp Psychiatry. 2009;40:317–28. 19176222 10.1016/j.jbtep.2009.01.002

[18] Fassbinder E, Schuetze M, Kranich A, Sipos V, Hohagen F, Shaw I, et al. Feasibility of group schema therapy for outpatients with severe borderline personality disorder in Germany: A pilot study with three year follow-up. Front Psychol. 2016;7. 10.3389/fpsyg.2016.01851 PMC5122742 27933020

[19] Arntz A, Jacob GA, Lee CW, Brand-De Wilde OM, Fassbinder E, Harper RP, et al. Effectiveness of predominantly group schema therapy and combined individual and group schema therapy for borderline personality disorder: A randomized clinical trial. JAMA Psychiat. 2022;79(4):287. 10.1001/jamapsychiatry.2022.0010 PMC8892362 35234828

[20] Arntz A. Group Schema Therapy for Borderline PD. Report to ZONMW: University of Amsterdam; 2017.

[21] Skewes SA, Samson RA, Simpson SG, van Vreeswijk M. Short-term group schema therapy for mixed personality disorders: a pilot study. Front Psychol. 2015;5. 10.3389/fpsyg.2014.01592 PMC4302795 25657631

[22] MIND. MIND kennisagenda [Internet]. Homepage. 2017 [cited 2022Jun28]. Available from: https://mindplatform.nl/nieuws/mind-kennisagenda-top-10-onderzoeksprioriteiten-vastgesteld. Accessed 28 June 2022.

[23] Landmann N, Kuhn M, Maier J-G, Spiegelhalder K, Baglioni C, Frase L, et al. REM sleep and memory reorganization: Potential relevance for psychiatry and psychotherapy. Neurobiol Learn Mem. 2015;122:28–40. 25602929 10.1016/j.nlm.2015.01.004

[24] Bamelis LL, Evers SM, Arntz A. Design of a multicentered randomized controlled trial on the clinical and cost effectiveness of schema therapy for personality disorders. BMC Public Health. 2012;12. 10.1186/1471-2458-12-75 PMC3305366 22272740

[25] de Klerk N, Abma TA, Bamelis LL, Arntz A. Schema therapy for personality disorders: A qualitative study of patients’ and therapists’ perspectives. Behav Cogn Psychother. 2017;45:31–45. 27573409 10.1017/S1352465816000357

[26] American Psychiatric Association. SCID-5-P Gestructureerd klinisch interview voor DSM-5 Persoonlijkheidsstoornissen. In: Nederlandse vertaling van Structured Clinical Interview for DSM-5® Personality Disorders (SCID-5-PD). Amsterdam: Boom; 2017.

[27] Arntz A, Jacob GA, Lee CW, Brand-de Wilde OM, Fassbinder E, Harper RP, et al. Effectiveness of predominantly group schema therapy and combined individual schema therapy for borderline personality disorder, a randomized clinical trial. JAMA Psychiatry. 2022;79:287-99. 10.1001/jamapsychiatry.2022.0010 PMC8892362 35234828

[28] Svartberg M, Stiles TC, Seltzer MH. Randomized, Controlled Trial of the Effectiveness of Short-Term Dynamic Psychotherapy and Cognitive Therapy for Cluster C Personality Disorders. Am J Psychiatry. 2004;161:810–7. 15121645 10.1176/appi.ajp.161.5.810

[29] Tjoa EEML, Muste EH. Handleiding groepschematherapie cluster C-persoonlijkheidsstoornissen. Bohn Staleu van Loghum; 2021.

[30] Farrell JM, Reiss N, Shaw IA. The schema therapy clinician’s guide. Hoboken: Wiley-Blackwell; 2014.

[31] Baljé A, Greeven A, van Giezen A, Korrelboom K, Arntz A, Spinhoven P. Group schema therapy versus group cognitive behavioral therapy for social anxiety disorder with comorbid avoidant personality disorder: study protocol for a randomized controlled trial. Trials. 2016;17:487. 27717405 10.1186/s13063-016-1605-9 PMC5055701

[32] van Vreeswijk MF, Broersen J. Kortdurende schemagroepstherapie: Cognitief gedragstherapeutische technieken. 2nd ed. Houten: Bohn Stafleu van Loghum; 2013.

[33] Baljé A et al. Psychometric properties of the AVPDSI (Unpublished manuscript).

[34] Tese S. Psychometric evaluation of the DPDSI. Thesis RINO Groep 2019.

[35] Verheul H. Psychometric evaluation of the OCPDSI. Thesis RINO Groep. 2020.

[36] Dreessen L, Arntz A. The personality disorder beliefs questionnaire (short version). Maastricht: author; 1995.

[37] Arntz A, Dreessen L, Schouten E, Weertman A. Beliefs in personality disorders: a test with the Personality Disorder Belief Questionnaire. Behav Res Ther. 2004;42:1215–25. 15350860 10.1016/j.brat.2003.08.004

[38] Rosenberg M. Society and the adolescent self-image. NJ. 1965.

[39] Franck E, de Raedt R, Barbez C, Rosseel Y. Psychometric properties of the Dutch Rosenberg self-esteem scale. Psychol Belg. 2008;48:25–35.

[40] Miskimins RW, Braucht GN. Description of the self: Rocky Mountain Behavioral Science Institute; 1971.

[41] Derogatis LR, Melisaratos N. The Brief Symptom Inventory: an introductory report. Psychol Med. 1983;13. 6622612

[42] de Beurs E, Zitman FG. De Brief Symptom Inventory (BSI): The reliability and validity of a brief alternative of the SCL-90. Maandblad Geestelijke Volksgezondheid. 2006;61:120–37.

[43] World Health Organization. World Health Organization disability assessment schedule WHODAS II. Geneva: World Health Organization; 2000.

[44] Üstün T, Kostanjsek N, Chatterji S, Rehm J. Measuring health and disability: Manual for WHO disability assessment schedule WHODAS 2.0. Geneva: World Health Organizaition; 2010.

[45] van Krugten FCW, Busschbach JJ, Versteegh MM, Hakkaart-van Roijen L, WBF B. The Mental Health Quality of Life Questionnaire (MHQoL): development and first psychometric evaluation of a new measure to assess quality of life in people with mental health problems. Qual Life Res. 2021;31:633-43. 10.1007/s11136-021-02935-w PMC8847188 34241821

[46] The EuroQol group. EuroQol-a new faciliEuroQol-a new facility for the measurement of health-related quality of lifety for the measurement of health-related quality of life. Health Policy. 1990;16:199–208. 10109801 10.1016/0168-8510(90)90421-9

[47] Long D, Polinder S, Bonsel J, Haagsma JA. Test–retest reliability of the EQ-5D-5L and the reworded QOLIBRI-OS in the general population of Italy, the Netherlands, and the United Kingdom. Qual Life Res. 2021;30:1–11. 10.1007/s11136-021-02893-3 PMC8481194 34075530

[48] Abdel-Khalek AM. Measuring happiness with a single-item scale. Soc Behav Personal Int J. 2006;34:139–50.

[49] Young JE, Brown G. Young schema questionnaire short form. New York: Cognitive Therapy Centre; 1998.

[50] Baranoff J, Oei TPS, Cho SH, Kwon S-M. Factor structure and internal consistency of the Young Schema Questionnaire (Short Form) in Korean and Australian samples. J Affect Disord. 2006;93:133–40. 16650482 10.1016/j.jad.2006.03.003

[51] Bamelis LLM, Renner F, Heidkamp D, Arntz A. Extended Schema Mode Conceptualizations for Specific Personality Disorders: An Empirical Study. J Pers Disord. 2011;25:41–58. 21309622 10.1521/pedi.2011.25.1.41

[52] Spoormaker VI, Verbeek I, van den Bout J, Klip EC. Initial validation of the SLEEP-50 questionnaire. Behav Sleep Med. 2005;3:227–46. 16190812 10.1207/s15402010bsm0304_4

[53] Arntz A, Venhuizen ASSM, Bachrach N, Groot IZ. Introversion Inventory (Unpublished manuscript). University of Amsterdam; 2021.

[54] Hoekstra HA, Ormel J, de Fruyt F. NEO PI-R, NEO-FFI Big Five Persoonlijkheids vragenlijsten: Handleiding. Lisse: Hogrefe; 1996.

[55] Baron-Cohen S, Sheelwright S, Skinner R, Martin CE. The Autism Spectrum Quotient (AQ): Evidence from Asperger syndrome/high functioning autism, males and females, scientists and mathematicians. J Autism Dev Disord. 2001;31:5–17. 11439754 10.1023/a:1005653411471

[56] Booth T, Murray AL, McKenzie K, Kuenssberg R, O’Donnell M, Burnett H. Brief report: An evaluation of the AQ-10 as a brief screening instrument for ASD in adults. J Autism Dev Disord. 2013;43:2997–3000. 23640304 10.1007/s10803-013-1844-5

[57] Allison C, Auyeung B, Baron-Cohen S. Toward brief “Red Flags” for autism screening: The short Autism Spectrum Quotient and the Short Quantitative Checklist in 1,000 cases and 3,000 controls. J Am Acad Child Adolesc Psychiatry. 2012;51:202–212.e7. 22265366 10.1016/j.jaac.2011.11.003

[58] Bernstein DP, Stein JA, Newcomb MD, Walker E, Pogge D, Ahluvalia T, et al. Development and validation of a brief screening version of the Childhood Trauma Questionnaire. Child Abuse Negl. 2003;27:169–90. 12615092 10.1016/s0145-2134(02)00541-0

[59] Thombs BD, Bernstein DP, Lobbestael J, Arntz A. A validation study of the Dutch Childhood Trauma Questionnaire-Short Form: Factor structure, reliability, and known-groups validity. Child Abuse Negl. 2009;33:518–23. 19758699 10.1016/j.chiabu.2009.03.001

[60] Schmand B, Bakker D, Saan R, Louman J. De Nederlandse Leestest voor Volwassenen: een maat voor het premorbide intelligentieniveau. Gerontologie en Geriatrie. 1991;22:15–9. 1877068

[61] DeRubeis RJ, Cohen ZD, Forand NR, Fournier JC, Gelfand LA, Lorenzo-Luaces L. The Personalized Advantage Index: Translating Research on Prediction into Individualized Treatment Recommendations. A Demonstration. PLoS One. 2014;9:e83875. 24416178 10.1371/journal.pone.0083875 PMC3885521

[62] Huibers MJ, Cohen ZD, Lemmens LH, Arntz A, Peeters FP, Cuijpers P, et al. Predicting optimal outcomes in cognitive therapy or interpersonal psychotherapy for depressed individuals using the personalized advantage index approach. PLoS One. 2015;10:e0140771. 26554707 10.1371/journal.pone.0140771 PMC4640504

[63] Versteegh MM, Vermeulen KM, Evers SM, De Wit GA, Prenger R, Stolk EA. Dutch tariff for the five-level Version of EQ-5D. Value Health. 2016;19:343–52. 27325326 10.1016/j.jval.2016.01.003

[64] Bouwmans C, de Jong K, Timman R, Zijlstra-Vlasveld M, van der Feltz-Cornelis C, Tan SS. Feasibility, reliability and validity of a questionnaire on healthcare consumption and productivity loss in patients with a psychiatric disorder (TiC-P). BMC Health Serv Res. 2013;13:1–9. 23768141 10.1186/1472-6963-13-217 PMC3694473

[65] Drummond MF, Sculpher MJ, Claxton K, Stoddart GL, Torrance GW. Methods for the economic evaluation of health care programmes: Oxford university press; 2015.

[66] Hakkaart-van Roijen L, van der Lindern N, Bouwmans C, Kanters T, Swan Tan S. Kostenhandleiding. 2016.

[67] Hendriks MRC, Al MJ, Bleijlevens MHC, van Haastregt JCM, Crebolder HFJM, van Eijk JTM, et al. Continuous versus intermittent data collection of health care utilization. Med Decis Making. 2013;33:998–1008. 23535608 10.1177/0272989X13482045

[68] Manca A, Hawkins N, Sculpher MJ. Estimating mean QALYs in trial-based cost-effectiveness analysis: the importance of controlling for baseline utility. Health Econ. 2005;14:487–96. 15497198 10.1002/hec.944

[69] Briggs AH, Wonderling DE, Mooney CZ. Pulling cost-effectiveness analysis up by its bootstraps: a non-parametric approach to confidence interval estimation. Health Econ. 1997;6:327–40. 9285227 10.1002/(sici)1099-1050(199707)6:4<327::aid-hec282>3.0.co;2-w

[70] Braun V, Clarke V. Using thematic analysis in psychology. Qual Res Psychol. 2006;3:77–101.

[71] Lincoln YS, Guba EG. Naturalistic inquiry. Beverly Hills: SAGE; 1985.

[72] Schreier M. Qualitative content analysis in practice. Thousand Oaks: SAGE; 2012.

[73] Schreier M. The Sage handbook of qualitative data collection. Londen: SAGE; 2018.

[74] Bastick E, Bot S, Verhagen SJW, Zarbock G, Farrell J, Brand-De Wilde O, et al. The development and psychometric evaluation of the Group Schema Therapy Rating Scale-Revised. Behav Cogn Psychother. 2018;46:601–18. 29370876 10.1017/S1352465817000741

