# Supplementary material for: Design of an RCT on cost-effectiveness of group schema therapy versus individual schema therapy for patients with Cluster-C personality disorder: the QUEST-CLC study protocol
Source: BMC Psychiatry. 2022 Oct 8;22:637. doi: 10.1186/s12888-022-04248-9 (PMC9548126; doi:10.1186/s12888-022-04248-9)
Supplement: Supplementary file 1 — Additional file 1. [file 12888_2022_4248_MOESM1_ESM.docx]

**Appendix A: Informed consent** (originally in Dutch, translated to English)

**CONFIDENTIAL**

**CONSENT STATEMENT**

The treatment of Cluster C personality disorders: a comparison of group schema therapy, individual schema therapy, and treatment as usual.

This form accompanies the written information you received about the study on the treatment of Cluster-C personality disorders. By signing this form, you declare that you have read and understood the participant information.

If you would like to receive further information about the study, you can contact the research coordinator of your institution, <<NAME>> <<CONTACT DATA>> or the researchers, Dr Nathan Bachrach, tel. 0492-848004, GGZ Oost Brabant, email N.Bachrach@ggzoostbrabant.nl and Prof Arnoud Arntz, University of Amsterdam, tel. 020-5256810 (secretariat), email a.r.arntz@uva.nl.

You can also address any complaints about this study to the contact person within the Ethics Committee of the Faculty of Social and Behavioural Sciences of the University of Amsterdam, Mr A. van Emmerik (telephone: 020-5256810; e-mail: a.a.p.vanemmerik@uva.nl).

[PARTICIPANT]

"I have read and understood the information and give permission for participation in the study and use of the data thus obtained to the University of Amsterdam and the other parties participating in the project as explained in the GST-CLC privacy statement of 18 March 2021. I reserve the right to withdraw this consent without giving any reason. I also retain the right to discontinue the research at any time.

*Signed in duplicate:*

Date:

............................................. …..............................

name of participant signature

[LOCAL REPRESENTATIVE]

“I have provided an explanation of the study. I declare my willingness to answer any further emerging questions on the study.”

Date:

….......................................... …..............................

Name of representative signature

*Other participating parties are: Maastricht University, GGZ Oost-Brabant - Helmond, GGZ Oost-Brabant - Oss, GGZ Oost-Brabant - Boxmeer, Emergis - Goes, IPGGZ Veendam, IPGGZ Groningen, PsyQ Amsterdam, PsyQ Utrecht, PsyQ Rotterdam, PsyQ Zaandam, Jolanda Messing (expert by experience), Natacha Maijs (expert by experience), Marjan Meelker-Lensen (expert by experience)*

**Appendix B: Organisational Structure and Responsibilities**

**Principal Investigator:**  prof. dr. Arnoud Arntz & dr. Nathan Bachrach

**Co-principal Investigator:**  dr. Raoul Grasman & prof. dr. Silvia Evers

**Junior Researchers:** Anne-Sophie Venhuizen & Iuno Groot

**Experience Experts:** Marjan Meelker & Natascha Maijs

**Site coordinators:**  Kasper Nikkels, Myrte Maarschalkerweerd, Susanne Dalmeijer, Joël

van Aalderen, Hinde de Lange, Renske Wichers, Agatha Hollander,

Bregje de Moor & Simone Walhout.

**Roles and Responsibilities**

The *Study Board* functions as the steering committee and is composed of all individuals listed above. Together they have final decision-making authority and share the ownership of the data. The study board meets in a low frequency; every six months or when needed.

The *Executive Committee* consists of the principal investigators and junior researchers and is responsible for the daily coordination of the trial. They oversee the implementation study, prepare the meetings, monitor the progress of the study and its legal, ethical and ICT aspects. The executive committee will perform site visits for every site during the inclusion period or when needed (e.g., high drop-out, low inclusion etc.). In addition, the junior researchers carry out audits on collected data to ensure the assessments are complete and on schedule.

The *Site Coordination Committee* is formed by the principal investigators, junior researchers, and all site coordinators. Monthly meetings are held to discuss the recruitment of patients, implementation of the study and to address potential difficulties.

**Appendix C: Dissemination of study results**

Results are communicated by publications, presentations and trainings. The following target groups can be distinguished:

*Clients, their relatives and the general public.*

Clients, their relatives and the general public are informed about the project and its outcomes in collaboration with client associations (Stichting Mind, Stichting Borderline), and the Dutch Knowledge Center Personality Disorders (Kenniscentrum Persoonlijkheidsstoornissen), with which we are connected through study board members Nathan Bachrach (clinician & researcher) and Jolanda Messing (experience expert). Press releases will be made and results will be shared through bulletins, newsletters and conferences for these groups. A website for the general public has also been made on which updates on study progress as well as the eventual study outcomes will be posted.

*Clinicians*.

Clinicians will be informed by presentations on (often large, n>800) (inter)national conferences, including of the VST (Vereniging SchemaTherapie (Dutch Association of Schema Therapy)), VGCT (Dutch Association of Behavioral and Cognitive Therapies), EABCT (European Association of Behavioural and Cognitive Therapies), ISST (International Society of Schema Therapy), ESSPD (European Society for the Study of PDs). Summarizing publications will be made for Dutch outlets (e.g., Tijdschrift voor Psychiatrie; Gedragstherapie). The protocols for GST and IST for Cluster-C PDs are already published in books and chapters. These will be updated based on results, especially results of the qualitative study among clients. We collaborate closely with Eelco Muste and Edith Tjoa, who developed the GST protocol. They will revise it when the study is finished. One of the authors current paper (Arnoud Arntz) developed the IST for Cl-C protocol, and will revise it when the study is finished. Trainings into GST and IST already exist, and will be revised based on the results of the study. Among our therapists are many trainers and supervisors, including of regional training institutes (RINO’s), which facilitates dissemination (train the trainers principle). Applicants have close connections to these RINO’s and to ST-specialized training institutes such as “Van Genderen Opleidingen BV”, “Psy-zo!”, and “Schematherapie in Bedrijf”, which are eager to offer training in evidence-based ST. We will lobby with the VST and ISST that the tested protocol(s) become the standard for application of ST for Cluster-C PDs, because accreditation of trainings depends on their recognition. AKWA, the Dutch organization for the care quality standards will be informed about the results, so that they can be integrated in the quality standard for PDs. Dutch guideline committees will be informed about the results. A byproduct of the study is the assessment of current severity of the pertinent PD. The severity interviews will be made available to clinicians for free, accompanied with short guidelines and summaries of psychometric properties.

*Managers of mental health institutes*.

Managers and heads of PD-treatment programs of large mental health institutes in the Netherlands will be informed about results by direct mailing. GST is already implemented at 10 participating sites, spread over the country, making these sites trendsetters, and influencing other treatment centers.

*Insurance committees*.

Insurance companies and notably their medical advisors will be informed about the results. Main findings will be presented in a meeting with the medical advisors of the insurance companies and implications discussed.

*Researchers*.

Dissemination to researchers is done by high impact scientific publications (open access) and presentations at conferences. The main applicant is regularly invited for keynotes.
